# Professor Jacobus Hendrikus Van't Hoff

**Published:** 1911-04-01

**Authors:** 


The death is announced from Berlin of Professor Jacobus Hendrikus van't Hoff, the eminent physicist and chemist, at the age of fifty-nine. Born at Rotterdam August 30, 1852, he was educated at the Technical School of Delft and at the University of Leyden, where he graduated in 1872 in mathematics and natural science. He next studied under KéKulé at Bonn, and took his doctor's degree at Utrecht in 1873. His next studying-place was Paris, where he worked under Pasteur and Würtz. In 1876 he was appointed professor at the Utrecht School of Veterinary Medicine, a year later was lector, and in 1878 professor at the University of Amsterdam. In 1896 he received a proposal from the Prussian Government, by which he was offered the membership of the Berlin Academy of Science and a professorship in the University. The laboratories of the institution were to be placed at his disposal without any obligation on his part to deliver lectures. In addition, his services were to be recompensed by a magnificent honorarium. The learned Dutchman accepted the offer, became a German citizen, and departed to add his lustre to that of the other members of the Berlin Academy. In 1901 he received the Nobel prize for chemistry, a worthy reward for a great scientific career. His death was the result of a tuberculous affection from which he had suffered for some time.

